# Supplementary material for: Indicators to distinguish symptom accentuators from symptom producers in individuals with a diagnosed adjustment disorder: A pilot study on inconsistency subtypes using SIMS and MMPI-2-RF
Source: PLoS One. 2019 Dec 30;14(12):e0227113. doi: 10.1371/journal.pone.0227113 (PMC6936836; doi:10.1371/journal.pone.0227113)
Supplement: Attachment — Submitted filename: Responses to Reviewers 2nd revision.docx [file pone.0227113.s002.docx]

**Reviewer 1**

The paper has been improved following the recommendations and I consider it suitable for publication.

**Thank you for reviewing our manuscript a second time, and to consider it suitable for publication.**

**Reviewer 2**

The authors have responded to most of my points. There are a few left.

**Thank you for reviewing our manuscript again. In the following points, you will find our answers to your suggestions. We have also highlighted our revisions in the text in yellow.**

Line 34; producers, and.

39; damage (or psychological/ psychiatric damage).

96. A study by.

96. scales'.

97; e.g.,

98. sensitive.

106. FBS-r.

108. FBS-r.

114 set."

120. have on SIMS.

126. judge's order,.

128. Rome, which.

133-4; 30% were a.

135; episodes, and.

135; originated from domestic.

141; e.g., .

149; e.g., .

153; did not.

154; i.e., .

157; times, while;

157; 23 cases involved mental;

157; reaching.

158; on the third. Table 1; still there;

219; Bonferroni not explained

237 and 253; letters still not clear; I did figure it out, though;

274; paper, we;

275; procedure, in which the;

277; sub-samples are used;

278; folds are then;

291; selection,".

291; means.

295; ran;

297; extraction is.

298; minimizes;

300; list.

318; the FI

319; note that.

326; Accentuator, .

327; based, .

328; model, called OneR, was run.

329; to highlight easily.

335; score emerged the;

336; identified.

337; it seems the results mentioned in the prior sentence are not provided. And would these results change anything in the discussion?

347; accentuators, and.

350; scores among participants.

428; feign.

448; vs. .

452; subjectivity; therefore, they.

**We have made all the requested changes, thank you.**

**Line 158. We have deleted Table 1 (“*Group’s composition according to the criteria set out”)* during the first revision, according to Reviewer1’s suggestion. The table that is now entitled Table 1 describes the “*Demographic Composition of the Three Research Groups*” (line 172).**

**Line 219. Regarding Bonferroni correction, SPSS automatically compute the *p* value after the adjustment. “*Confidence interval adjustment. Select least significant difference (LSD), Bonferroni, or Sidak adjustment to the confidence intervals and significance*” (IBM SPSS Advanced Statistics 26, p.15). Moreover, we have added this sentence in “Statistical Analysis Section”: “*The Bonferroni correction was applied to adjust confidence intervals; SPSS-25 software (SPSS Inc., Chicago, IL) automatically corrected the p-value for the number of comparisons.*”**

**Line 337. To greater clarity, we have rephrased the sentence as follow: “*According to the aforementioned classification rules, indeed, OneR identified cut-off for the SIMS score (i.e., 13.5, 18.5, and 34.5) to distinguish symptom producers from symptom accentuators and consistent participants.*”**
